# The effect of a transforaminal epidural injection in patients with lumbar disc herniation is not correlated with the presence of type II modic changes

**DOI:** 10.1016/j.bas.2025.104222

**Published:** 2025-02-24

**Authors:** E.J.A. Verheijen, N.R.E. van der Vlist, E.C. Bartels, O.B.H.A.M. van Haagen, C.L.A. Vleggeert-Lankamp

**Affiliations:** a Department of Neurosurgery, Leiden University Medical Center, Leiden, the Netherlands; b Department of Neurosurgery, Spaarne Hospital, Hoofddorp, Haarlem, the Netherlands; c Department of Anesthesiology, Spaarne Hospital, Hoofddorp, Haarlem, the Netherlands

**Keywords:** Lumbar disc herniation, Lumbar radicular pain, Transforaminal epidural steroid injection, Modic changes, Vertebral endplate changes, Patient-perceived recovery

## Abstract

**Introduction:**

Transforaminal epidural steroid injections (TEI) have been suggested to alleviate symptoms in patients with lumbar disc herniation (LDH) through its anti-inflammatory effect. However, treatment effect varies among patients and reliable predictors are lacking. Modic changes (MC) are also associated with inflammatory processes and, therefore, we hypothesize that MC may be correlated with outcome after TEI.

**Research question:**

To investigate the correlation between the presence of MC at the level of LDH and the effect of TEI.

**Material and methods:**

Patients with unilateral lumbar radiculopathy secondary to LDH undergoing TEI were included. MC was graded by two independent assessors. Outcome measures included leg pain, back pain, disability and patient-received recovery at baseline, 30 min, 2 weeks and 6 weeks after treatment. Multivariate analysis was performed for all outcomes and for dichotomized scores using a cutoff of ≥30% improvement. A p-value of ≤0.05 was considered statistically significant.

**Results:**

A total of 88 patients were included of whom 52.3% demonstrated MC. The vast majority was classified as type II (94%). The presence of MC was not correlated with any outcome measure when correcting for age, gender, duration of symptoms and the use of analgesics, nor for dichotomized scores.

**Discussion and conclusion:**

The findings indicate that type II MC is not associated with outcome within six weeks after TEI. Therefore, type II MC cannot be used as a predictor for TEI outcome. Future studies should include longer follow-up and investigate the correlation between the type of MC and the effect of TEI.

## Highlights

• Type II Modic changes are the most prevalent of all three types
• Modic changes do not correlate with the effect of TEI on pain
• Modic changes do not correlate with the effect of TEI on disability or recovery

## 1 Introduction

Low back pain and unilateral lumbar radiculopathy are common spinal problems associated with disc herniation in the lumbar spine (LDH) (Waldman and Waldman, 2019). Lifetime prevalence rates of up to respectively 80% and 43% have been reported for these conditions (Hoy et al., 2012; Konstantinou and Dunn, 2008) and patients can experience a severe decline of physical functioning as a result. Although symptoms usually spontaneously diminish with time, the period until full recovery can be very debilitating when the pain is insufficiently alleviated with oral analgesics.

In these cases, additional treatment with a Transforaminal Epidural Steroid Injection (TEI) can help to reduce pain symptoms. It is assumed that radicular pain is caused by a combination of mechanical compression, inflammatory reactions and immunological processes affecting the lumbar nerve root (Stafford et al., 2007). Epidural injections with a corticosteroid aim to reduce the inflammation around the nerve root by limiting nerve root oedema, blocking prostaglandin synthesis and altering the conduction of nociceptive C-fibres (McLain et al., 2005; Collighan and Gupta, 2009; Johansson et al., 1990; Kantrowitz et al., 1975). Therefore, this minimally invasive treatment may reduce radicular symptoms and enable the patient to maintain physical functionality, restore quality of life, and, if satisfactorily effective, obviate the need for neurosurgical intervention.

Despite the potential benefits, treatment results with TEI in patients with LDH vary considerably and, therefore, factors that can differentiate between responders and non-responders to TEI would be valuable to achieve personalized treatment strategies. Vertebral end plate changes, or Modic changes (MC), have been suggested to reflect the presence of inflammatory processes and are associated with disc herniation and less favorable outcomes after surgery (Dudli et al., 2017, 2018, 2022; Viswanathan et al., 2020; Heggli et al., 2023; Modic et al., 1988; Albert et al., 2008; Djuric et al., 2019). It is postulated that the absence of MC is associated with less radicular pain and better clinical outcome, whereas the presence of MC aggravates radicular symptoms (Djuric et al., 2019). Hence, we hypothesize that in patients with MC at the level of disc herniation the inflammatory component may have a larger contribution to the radicular symptoms of the patient and, therefore, TEI with anti-inflammatory medication may be more effective. To the best of our knowledge, only two studies have assessed this association before, but demonstrated contradictory results (Lee et al., 2013; Peterson et al., 2014).

Therefore, in this prospective cohort study, we aim to determine the effect of TEI on various clinical outcomes in patients with unilateral radiculopathy secondary to LDH stratified by the presence or absence of MC.

## 2 Material and Methods

### 2.1 Patient inclusion

Patients were included that participated in the POTEISS study, an ongoing large prospective cohort study aimed at developing a prediction model for TEI success for patients with lumbar radiculopathy due to LDH or degenerative spinal stenosis. Patients who were referred by the neurologist or neurosurgeon to the outpatient pain clinic in the Spaarne Hospital, Hoofddorp and Haarlem, the Netherlands, for treatment with TEI were eligible for inclusion in this study if clinical findings were in accordance with Magnetic Resonance Imaging (MRI) examination. Patients younger than 18 years, with severe multisegmental spinal degeneration, anatomical abnormalities that may complicate TEI treatment technically (e.g. severe scoliosis), a history of lower back surgery at the same lumbar level and side, previous TEI treatment for their current episode of lumbar radiculopathy, active malignancy or infectious disease, the use of immunosuppressive drugs or systemic corticosteroids in the preceding 3 months, pregnancy, circumstances preventing treatment with TEI (e.g. use of anticoagulants that cannot be temporarily discontinued) or a major language barrier were excluded. Patients were contacted by phone one week before their scheduled TEI appointment and informed about the study. If the patient was willing to participate, they were contacted again two days before treatment for informed consent and to fill in baseline questionnaires.

### 2.2 Data collection

Demographical data were collected at baseline and processed using Castor EDC, a web-based data capture platform (Ciwit B.V., Amsterdam, The Netherlands). Clinical data included the average leg pain score for the past seven days (Numerical Rating Scale (NRS)), average back pain for the past seven days (NRS) and Oswestry Disability Index (ODI) for physical functionality and were gathered at baseline, 2 and 6 weeks after TEI treatment. In addition, for leg and back pain NRS scores at 30 min after the procedure were collected by one of the outpatient pain clinic nurses. Furthermore, patient perceived recovery (GPE) was measured at 2 and 6 weeks follow-up on a 7-point Likert scale.

### 2.3 MC grading

**Fig. 1:**
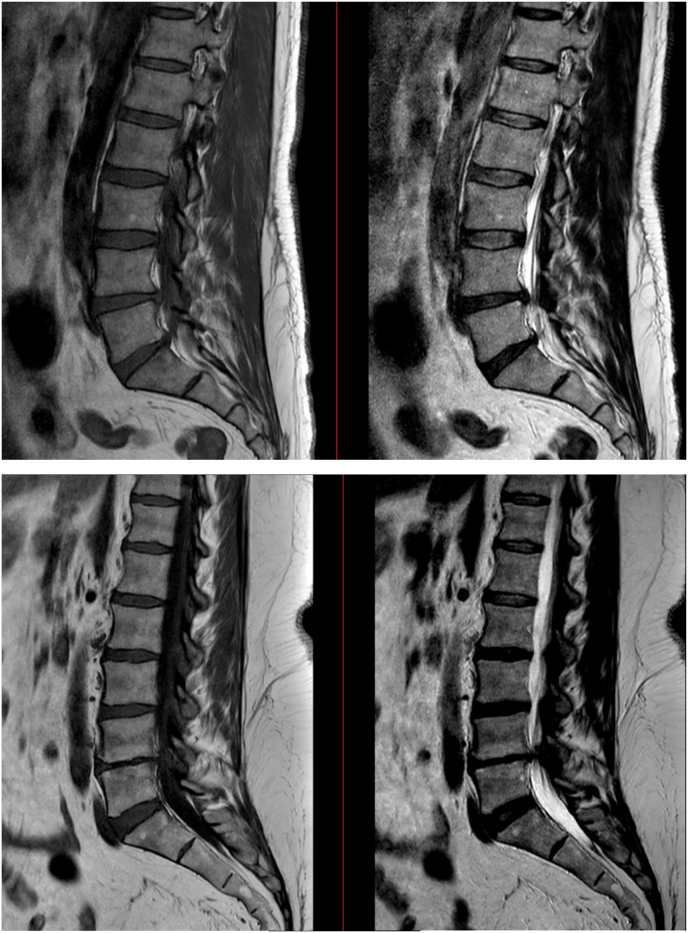
Disc herniation at L4-5. Upper panel: Sagittal T1-and T2-weighted MRI images showing normal bone marrow intensity and absence of Modic changes. Lower panel: Sagittal T1-and T2-weighted MRI images showing hyperintense areas around the L4-5 disc, indicating type II Modic changes.

For each patient, the T1-and T2-weighted sagittal MRI scans were assessed by two independent readers (CVL and EV) to determine the level of LDH, presence or absence, type (I, II or III) and severity of MC (mild, moderate or severe) at the same level of the LDH. Grading of MC was performed according to the original classification by Modic et al.: type 1 changes were hypointense on T1-weighted imaging (T1WI) and hyperintense on T2-weighted imaging (T2WI), type 2 changes were hyperintense on T1WI and isointense to hyperintense on T2WI, and type 3 changes were hypointense on both T1WI and T2WI (Modic et al., 1988). If a mixture of MC types was shown, it was categorized according to the most abundant type at the LDH level. The severity of MC was determined by the degree of endplate involvement: mild (<25%), moderate (25–75%) and severe (>75%). In case of discrepancies, consensus was reached through discussion. An example of patient without MC and with MC is shown in Fig. 1.

### 2.4 Treatment procedure

Administration of TEI was performed by an anesthesiologist who determined the treatment level based on clinical findings and radiological imaging. The patient lay in a prone position on the table with a support cushion underneath the abdominal region to reduce the natural lordosis. The skin was sterilized with chlorhexidine and locally anaesthetized with 1 mL 1% lidocaine. Under fluoroscopic image guidance the needle was placed via a transforaminal approach in close proximity of the nerve root. Contrast agent was injected to confirm correct positioning of the needle. In accordance with current Dutch anesthesiologic guidelines, injections at L3 and below contained 1,5 ml lidocaine 2% and 40 mg methylprednisolone acetate and injections above L3 contained 1,5 ml lidocaine 1% and 10 mg dexamethasone to prevent vascular occlusion (Dietrich et al., 2015). After the procedure, the patient was monitored for 30 min at the recovery room.

### 2.5 Sample size

The minimal sample size required to assess the correlation between the presence of Modic changes and the effect of TEI was calculated using a two-tailed α of 0.05 and β of 0.2. We aimed to include a sufficient number of patients that would allow to determine whether a correlation coefficient indicating low correlation (r = 0.3) differed from zero. Therefore, a total sample size of at least 85 patients was necessary (Hulley et al., 2013).

### 2.6 Data analysis

Statistical analysis was performed using SPSS version 29 (IBM Corp., Armonk, New York, USA). Demographical data were analyzed using descriptive statistics for patients with and without MC separately using appropriate tests depending on the normality of distribution. Linear mixed models were employed to assess the association between the presence of MC (as main effect) and change scores in leg pain, back pain and ODI at follow-up correcting for baseline differences and multiple testing (Holm-Bonferroni correction). Age, gender, duration of symptoms and the use of pain medication at baseline were used as covariates. Additionally, linear mixed models analysis was conducted using change scores expressed in percentages compared to baseline and generalized estimating equations analysis for dichotomized scores for leg pain, back pain, ODI (a decrease of 30% or more compared to baseline was categorized as ‘success’) and patient perceived recovery (a score of 1 or 2 was considered as ‘success’). Loss-to-follow-up was reported for every outcome variable at the various time points and accounted for using statical analysis methods that can handle missing data. A p-value <0.05 was considered to be statistically significant.

## 3 Results

A total of 88 patients was included in this study with 52.3% (n = 46) showing MC at the level of LDH. Type II MC was most common with a prevalence of 93.5% (n = 43). At baseline, patients with MC had less leg and back pain, while ODI did not differ. Other demographic variables were not different between groups (Table 1).

**Table 1:** Baseline characteristics for patients with and without MC at the level of LDH. Continuous variables are presented as mean ± standard deviation; categorical variables are presented in number (percentage).

Variable	Modic changes (n = 46)	No Modic changes (n = 42)	P value
Age (yrs)	56.9 ± 13.6	50.6 ± 17.2	0.058
Gender (male)	20 (43.5%)	21 (50.0%)	0.540
BMI (kg/m^2^)	25.9 ± 3.0	26.0 ± 3.6	0.936
Duration of symptoms (wks)	47.9 ± 70.9	32.0 ± 52.4	0.239
Smoking (yes)	10 (21.7%)	13 (31.0%)	0.326
Alcohol usage			0.254
None	13 (28.3%)	19 (45.2%)	
1–6 units/wk	24 (52.2%)	17 (40.5%)	
>6 units/wk	9 (19.6%)	6 (14.3%)	
Side of leg pain (left)	24 (52.2%)	21 (50.0%)	0.839
Use of pain medication (yes)	39 (84.8%)	36 (85.7%)	0.902
Paracetamol	34 (73.9%)	30 (71.4%)	
NSAID	20 (43.5%)	18 (42.9%)	
Morphine	17 (37.0%)	12 (28.6%)	
Physical therapy (yes)	12 (26.1%)	16 (38.1%)	0.227
Level of LDH			0.408
L2-L3	1 (2.2%)	1 (2.4%)	
L3-L4	4 (8.7%)	5 (11.9%)	
L4-L5	21 (45.7%)	25 (59.5%)	
L5-S1	20 (43.5%)	11 (26.2%)	
Type of Modic change			
Type I	2 (4.3%)		
Type II	43 (93.5%)		
Type III	1 (2.2%)		
Severity of Modic change			
Mild	28 (60.9%)		
Moderate	15 (32.6%)		
Severe	3 (6.5%)		
Baseline leg pain	5.6 ± 1.9	6.9 ± 1.3	<0.001
Baseline back pain	4.0 ± 2.4	5.2 ± 2.2	0.019
Baseline ODI	42.1 ± 18.4	43.0 ± 15.8	0.806

**Fig. 2:**
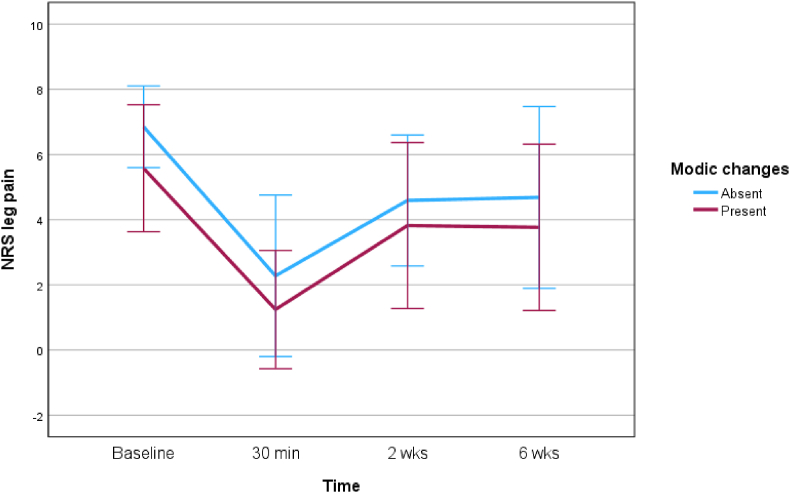
Comparison of the effect of TEI on leg pain between patients with MC and without MC at the level of LDH.

**Fig. 3:**
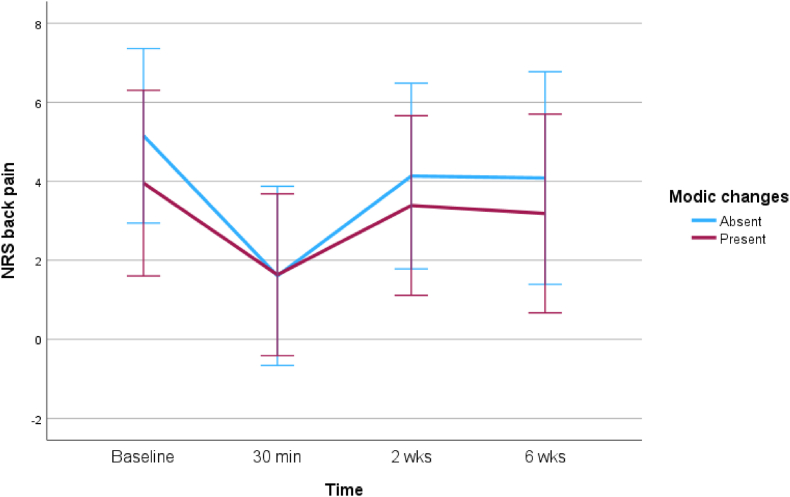
Comparison of the effect of TEI on back pain between patients with MC and without MC at the level of LDH.

**Fig. 4:**
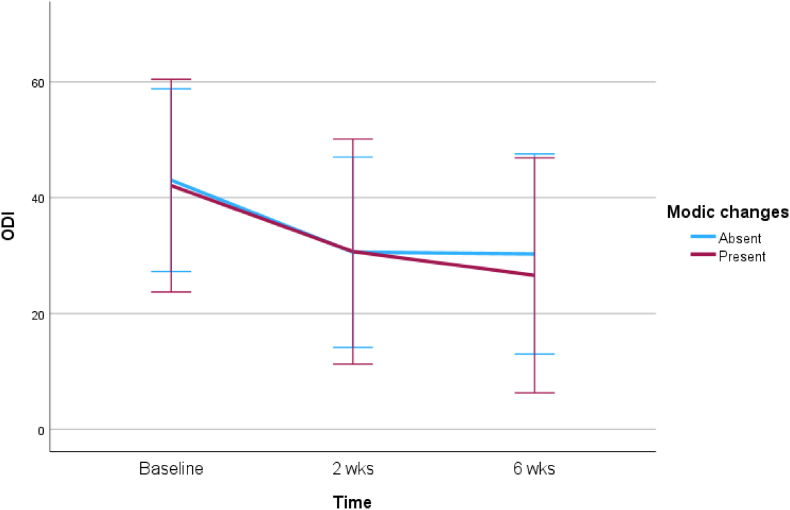
Comparison of the effect of TEI on functional status between patients with MC and without MC at the level of LDH.

Leg pain, back pain and ODI scores decreased during 6 weeks for both groups (Fig. 2, Fig. 3, Fig. 4). Data were missing for some patients depending on the outcome variable and follow-up time point (Table 2).

**Table 2:** Missing data for each outcome variable at all follow-up time points.

FU	NRS leg pain	NRS back pain	ODI	GPE
30 min	19/88 (21.6%)	21/88 (23.9%)	–	–
2 wks	6/88 (6.9%)	7/88 (8.0%)	7/88 (8.0%)	8/88 (9.1%)
6 wks	13/88 (14.8%)	14/88 (15.9%)	14/88 (15.9%)	15/88 (17.0%)

GPE: Global Perceived Effect (recovery); NRS: Numerical Rating Scale; ODI: Oswestry Disability Index.

Changes in leg pain scores compared to baseline were not associated with the presence or absence of MC when controlling for age, gender, duration of symptoms and the use of analgesic medication (estimate of the fixed effect −0.429; standard error 0.315; 95% CI -1.051, 0.194; p-value 0.176). When converting absolute change scores to changes expressed as a percentage of the baseline score, no association with MC was demonstrated either (estimate −3.209; SE 5.185; 95% CI -7.021, 13.43; p-value 0.537). Back pain was neither associated with MC at the level of LDH for absolute change scores (estimate −0.471; SE 0.353; 95% CI -1.168, 0.225; p-value 0.184) nor percentual changes scores (estimate 4.483; SE 12.310; 95% CI -19.872, 28.838; p-value 0.716). ODI representing the patient's functional status was not associated with MC for both absolute and percentual change scores compared to baseline (estimate 0.697; SE 2.236; 95% CI -3.724, 5.118; p-value 0.756 and estimate 5.292; SE 5.608; 95% CI -5.793, 16.377; p-value 0.347).

All continuous outcome variables were dichotomized using a cut-off of a 30% or more decrease compared to baseline to indicate ‘success’ after TEI treatment. The presence of Modic changes was not associated with the number of patients categorized as ‘success’ based on leg pain scores (estimate 0.083; SE 0.3022; 95% CI -0.509, 0.676; p-value 0.783), back pain scores (estimate −0.195; SE 0.3247; 95% CI -0.832, 0.441; p-value 0.548), nor scores for functional status (estimate 0.084; SE 0.3671; 95% CI -0.636, 0.052; p-value 0.820). In addition, dichotomized patient perceived recovery was neither associated with the presence or absence of MC (estimate −0.186; SE 0.3936; 95% CI -0.958, 0.585; p-value 0.636) (Table 3).

**Table 3:** Number of patients (percentages) that demonstrated a decrease of 30% or more compared to baseline for leg pain, back pain and ODI or considered their recovery ‘successful’ after treatment with TEI.

	NRS leg pain		NRS back pain		ODI		GPE	
	**MC**	**No MC**	**MC**	**No MC**	**MC**	**No MC**	**MC**	**No MC**
30 min	35/40 (87.5%)	22/29 (75.9%)	28/40 (70.0%)	20/27 (74.1%)	–	–	–	–
2 wks	22/43 (51.2%)	20/39 (51.3%)	19/43 (44.2%)	16/38 (42.1%)	19/43 (44.2%)	16/38 (42.1%)	12/43 (27.9%)	12/37 (32.4%)
6 wks	18/37 (48.6%)	21/37 (56.8%)	13/38 (34.2%)	19/36 (52.8%)	21/38 (55.3%)	19/36 (52.8%)	16/38 (42.1%)	16/35 (45.7%)

GPE: Global Perceived Effect (recovery); NRS: Numerical Rating Scale; ODI: Oswestry Disability Index.

## 4 Discussion

This study has demonstrated that the presence or absence of Modic changes at the level of disc herniation is not associated with changes in leg pain, back pain, physical functionality nor patient-perceived recovery within six weeks after TEI treatment. Conclusions should be narrowed to type II MC, since 94% of patients with MC had type II MC. In both groups leg pain and back pain decreased over time, and physical functionality increased, with the largest change between baseline and two weeks after treatment, but differences between groups were not statistically significant. These results did not change when considering change scores expressed in percentages or when dichotomized to ‘success’ or ‘non-success’.

We hypothesized that in patients with MC at the level of LDH inflammatory processes may play a larger role and aggravate radicular symptoms causing TEI to be more effective. However, our results demonstrated no correlation between the effect of TEI and presence of MC. Possibly, MC may not be a marker for nerve root inflammation although it has been associated with disc herniation and less favorable outcomes after disc surgery (Albert et al., 2008; Djuric et al., 2019). More likely, the correlation may have been obscured by the preponderance of MC type II among our study population. Although both type I and type II, the most prevalent types of MC, are associated with inflammatory changes, it is thought that the pathophysiological mechanisms are different. Type II is assumed to have a more chronic character, which fits to the mean duration of symptoms of 32 and 48 weeks in the studied patient groups. It is possible that TEI is less effective in those patients, which might explain the lower percentage of responders among our patients compared to Joswig et al. (48.6–56.8% vs. 66.7%), who reported a mean duration of symptoms of 14.8 weeks (Joswig et al., 2016). Therefore, the effect of TEI may be more pronounced when comparing patients without MC to those with MC type I, as the latter represents a more acute course of symptoms. Furthermore, it is possible that the inflammatory processes related to MC type II may take time to respond to the injection, and these effects may not be noticeable in the early post-injection period, thus requiring longer follow-up.

Our findings agree with the results demonstrated by Lee et al. (2013). Among a set of 149 patients that received up to three transforaminal injections for lumbar radiculopathy caused by LDH, MRI parameters were investigated in a retrospective cohort study. Only patients with the very best or very worst outcome after treatment were included. 13% of patients had MC, 74% of them demonstrated type II MC, and the majority of the patients had symptoms for less than one month. The authors found no correlation between the presence or absence of Modic changes and response after TEI measured by the Visual Analogue Scale (VAS) and a 5-point self-satisfaction scale. However, for their analyses, they did not specify the average time between treatment and outcome assessment. Contrastingly, Peterson et al. did observe a correlation between the presence of Modic changes and pain severity after TEI in their prospective cohort study (Peterson et al., 2014). They included a total of 346 patients; 57% of them had MC and 67% of these patients demonstrated type II MC. They found that patients with MC (type I and II combined) reported significantly lower levels of pain reduction compared to patients without MC at one month follow-up (p = 0.04), but not for shorter follow-up nor for dichotomized Patient's Global Impression of Change (PGIC). The percentage of patients that considered themselves improved, 1 month after TEI (40–50%) was comparable to our results (42–46% after 6 weeks). However, their methods were slightly different as patients were included presenting with MC at any level and not necessarily at the level of disc herniation. They hypothesized that there could be a difference in outcomes between patients with and without MC, which has been associated with low back pain, as patients with MC would have an additional pain generator. Yet, in our opinion, TEI is aimed at relieving radicular symptoms rather than back pain, and the authors did not specify whether the NRS pain scores patients reported concerned leg pain or back pain. Moreover, patients could receive injections for multiple nerve roots and statistical analyses did not correct for any confounders. In addition, very few baseline characteristics were presented for the patient groups with and without MC as reliable information on socio-demographic factors was not available rendering a multivariate analysis in their study infeasible. Finally, 65 patients in this cohort demonstrated type I MC, which may have contributed to a different outcome.

The results from our study are strengthened by the strict in- and exclusion criteria applied during inclusion of patients. Only patients with unilateral radiculopathy and clinical symptoms that were concordant with radiological findings on MRI, were eligible to participate. Furthermore, treatment was limited to a single fluoroscopy-guided injection and no repeat injections were performed during follow-up. This allowed for a more appropriate evaluation of TEI effect compared to studies that included patients with bilateral symptoms, without radiological confirmation of LDH, offered bilateral or multi-level injections at baseline or repeat injections during follow-up. Moreover, the prospective nature of this study allowed for the use of validated outcome instruments to systematically collect data on the effect of TEI treatment. Finally, the dichotomization of NRS scores for leg pain was performed using a cut-off that has been defined by an international consensus group of experts to obtain success data (Ostelo et al., 2008). The use of dichotomized data can provide other insights on the effect of TEI, since it is possible that the true effect is obscured using mean data from a continuous variable (Ghahreman et al., 2010).

The current study has a few limitations. First, the total number of patients included was small since this was a subset of a larger cohort study. For some patients outcome data was missing which reduced the number of complete cases for our analyses. Furthermore, the vast majority of patients with Modic changes demonstrated type II which precluded a sub-analysis of the type of MC on the effect of TEI.

Future studies should include a larger sample size to obtain a higher statistical power and to investigate the correlation between MC type and the effect of TEI. In addition, more patients with a shorter duration of symptoms should be included to determine whether MC type I is more prevalent in this group and if the outcome after TEI differs compared to those with MC type II and those without MC.

The findings from our study indicate that there is no correlation between the presence or absence of type II Modic changes at the same level of LDH and outcome after TEI on the short term. Therefore, clinicians should not consider the presence of type II MC to be a contra-indication for treatment with TEI.

## Funding

This research did not receive any specific grant from funding agencies in the public, commercial, or not-for-profit sectors.

## Declaration of competing interest

The authors declare that they have no known competing financial interests or personal relationships that could have appeared to influence the work reported in this paper.
